# Influence of native thin filament type on the regulation of atrial and ventricular myosin motor activity

**DOI:** 10.1016/j.jbc.2024.107854

**Published:** 2024-10-05

**Authors:** Emrulla Spahiu, Petra Uta, Theresia Kraft, Arnab Nayak, Mamta Amrute-Nayak

**Affiliations:** Institute of Molecular and Cell Physiology, Hannover Medical School, Hannover, Germany

**Keywords:** cardiac contraction, atrium, ventricle, actomyosin, tropomyosin-troponin complex

## Abstract

Ca^2+^-mediated activation of thin filaments is a crucial step in initiating striated muscle contraction. To gain mechanistic insight into this regulatory process, thin filament (TF) components and myosin motors from diverse species and tissue sources are often combined in minimal *in vitro* systems. The contribution of tissue-specific TF composition with native myosin motors in generating contraction speed remains unclear. To examine TF-mediated regulation, we established a procedure to purify native TFs (nTF) and myosin motors (M-II) from the same cardiac tissue samples as low as 10 mg and investigated their influence on gliding speeds and Ca^2+^ sensitivity. The rabbit atrial and ventricular nTFs and M-II were assessed in *in vitro* nTF motility experiments under varying Ca^2+^ concentrations. The speed-pCa relationship yielded a maximum TF speed of 2.58 μm/s for atrial (aM-II) and 1.51 μm/s for ventricular myosin (vM-II), both higher than the respective unregulated actin filament gliding speeds. The Ca^2+^ sensitivity was different for both protein sources. After swapping the nTFs, the ventricular TFs increased their gliding speed on atrial myosin, while the atrial nTFs reduced their gliding speed on ventricular myosin. Swapping of the nTFs decreased the calcium sensitivity for both vM-II and aM-II, indicating a strong influence of the thin filament source. These studies suggest that the nTF-myosin combination is critical to understanding the Ca^2+^ sensitivity of the shortening speed. Our approach is highly relevant to studying precious human cardiac samples, that is, small myectomy samples, to address the alteration of contraction speed and Ca^2+^ sensitivity in cardiomyopathies.

During a cardiac cycle, in a systolic phase, large blood volumes in the ventricular chamber are ejected and distributed throughout the body through powerful ventricle contraction, while atrial contraction assists in ventricle chamber refilling during diastole. Myosin II is a major motor protein responsible for atrial and ventricular contractions. Each myosin holoenzyme is a hexamer comprised of two heavy chains (HC) and four light chains (LC). In higher mammals, such as rabbits, pigs, humans, the ventricles express predominantly the β isoform of the myosin heavy chain (β-MyHC) while the atria express the α isoform (α-myosin heavy chain, α-MyHC). Despite having a 91% identical amino acid sequence (1), the α and β myosin isoforms display distinct biochemical and mechanical properties (2). The α-and β-MyHCs are expressed from different genes, *MHY6* and *MHY7*, respectively. Each HC N-terminal globular motor domain has binding sites for ATP and actin and is followed by helical domain which associate with two LCs. Together, the helical domain and LCs form the so-called neck region or lever arm of the myosin. The C-terminal HC neck domain is followed by the S2 domain and a long α-helical tail domain. The S2 and the tail domain self-assemble to form a coiled-coil dimer. Specific light chains associate with the α-MyHC and β-MyHC isoforms. Atrial specific myosin light chain 1 and light chain 2 (MLC1a and MLC2a) are bound to α-MyHC, and ventricular specific MLC1v and MLC2v are bound to β-MyHC. The light chains are reported to play a structural role in stabilizing the lever arm as well as modulating the motor activity (3, 4, 5).

Myosin’s endogenous partner in the generation of myocardial contraction is the thin filament, comprising filamentous actin decorated with the regulatory proteins tropomyosin (Tm) and trimeric troponin (Tn) complex with troponin-T (TnT), troponin-C (TnC), and troponin I (TnI). Cardiac contraction is regulated by Ca^2+^, whereby myosin thick filaments slide past thin filaments in the sarcomere (6). Ca^2+^ binds to the troponin complex on the thin filament to initiate and regulate the association between thick and thin filaments (7). The rise and fall in the intracellular Ca^2+^ thus modulate the contraction-relaxation cycle (8, 9).

In the regulatory Tm–Tn complex, Tm is an α-helical coiled-coil protein that spans over seven G-actin subunits on one of the two long-pitch helical strands of the actin filament, forming a continuous helical cable along the actin filament. In a heterotrimeric troponin complex, each Tn plays a distinct role. For example, TnC is the Ca^2+^ binding subunit, and TnI is an inhibitory subunit with the C terminus partly extending along the Tm while the N terminus interacts with TnC and TnT. TnI is known to inhibit actomyosin ATPase activity, and TnT binds to Tm. Tn complex binding at a distinct position of Tm creates the periodicity of about 38.5 nm corresponding to the actin strand of seven subunits, thus the actin:Tm:Tn stoichiometry of 7:1:1 is maintained (8, 10, 11, 12, 13, 14).

Although there is no ambiguity about the importance of Ca^2+^ in regulating the productive actomyosin interactions through Tm–Tn complex on the actin filament, the sequence of events triggering this process is described by two models. According to the steric blocking model, Tm displacement correlates with the existence of “OFF” or inactive and “ON” or active states of the TFs (15, 16). Accordingly, at low Ca^2+^ concentration, the Tm cable blocks myosin-binding sites, whereas at high Ca^2+^ concentration, Tm moves azimuthally around actin to expose these sites (17, 18). The 3-state model proposed three TF structural states, that is, blocked, closed, and open, the states corresponding to relax, active, and strong myosin-bound configurations, respectively (19). Accordingly, at low Ca^2+^ concentration, the Tn complex is tightly bound to Tm through TnT subunits and actin through TnI subunits and positions the Tm in a blocked state, where Tm sterically hinders myosin heads from binding to actin (20). Transient weak interactions between myosin and actin are proposed to be possible in a blocked state. When Ca^2+^ is bound to the TnC subunit, reorganization of the Tn subunits makes binding between Tm and Tn weaker (21), displacing Tm from actin in a closed state, where myosin heads can weakly interact with actin. During the transition from weak to strong actin-myosin binding, the myosin head shifts Tm to an open state, exposing adjacent myosin-binding sites on actin for further myosin binding (19, 22, 23). Recent study from Risi *et al.* proposed two different structural states of TFs at high Ca^2+^ concentration, that is, partially active and fully active TFs. In partially active state, TnI C-terminal domain weakly interacts with actin-Tm, while in fully active state, TnI C-terminal domain was found disordered/detached from actin-Tm (24).

The regulatory position of Tm on thin filaments can be influenced by both Ca^2+^ binding to TnC and myosin binding to actin, that is, Ca^2+^ or myosin alone does not induce a full TF activation, but collective action leads to unblocking the myosin-binding sites along the actin filament (25, 26).

Native thin filaments (nTFs) isolated from cardiac or skeletal muscle tissue and reconstituted TFs are generally used in *in vitro* studies to understand the basic principles of thin filament activation and its relation to myosin-dependent mechanical outcome. Besides, this experimental approach is employed to investigate cardiomyopathy-causing mutations in the myosin complex that are known to alter contractile properties and Ca^2+^ sensitivity. Synthetic TFs are reconstituted from actin, Tm, and Tn. Here the Tm–Tn complex is either individually purified from tissue and then combined with the actin filaments (27), or each individual component is expressed and then reconstituted. In some studies, reconstituted TFs were found to be less Ca^2+^ sensitive than nTFs. This difference in Ca^2+^ sensitivity likely arises due to the differences in nTF components, their posttranlational modifications (PTMs) and/or the different structures of native and synthetic filaments, although no major structural differences were noted for reconstituted *versus* native TFs (8).

With currently available standard methods, the isolation and purification of native thin filaments from tissue requires several grams of starting tissue material and the experimental protocol takes several days to produce sufficient quantities of regulated thin filaments. Often, the TFs and myosins are from different muscle sources and even from different species, that is, this combination of myosin isoforms and TFs as functional partners may not co-exist in a physiological setting. To overcome these limitations and address important questions related to the function of native thin filaments, we developed a procedure for extracting native myosin and isolating native thin filaments from one-and-the same cardiac muscle tissue sample. To our knowledge, *in vitro* studies with cardiac tissue–specific combinations of native thin filaments and myosin motors were not reported. We further investigated the atrial and ventricular thin filaments for their possible similarities or differences in their activation, what was not compared before. Rather, the cardiac thin filaments were considered to be similar in their properties, and only myosin-specific chemomechanical features remained the main focus for evaluations of the actomyosin cross-bridge cycle in earlier studies.

Our new procedure to isolate TFs and myosin from the same tissue allowed us to directly determine the contribution of the thin filaments to myosin-driven gliding speeds. Usage of tissue-specific myosin isoform composition (α and β) and native thin filaments allowed us to probe the extent to which the atrial and ventricular mechanical characteristics are determined by the myosin isoform expressed and the contribution of thin filaments in this process, revealing the collective function of this multicomponent system directly in relation to their physiological roles.

## Results

To investigate thin filament activation and Ca^2+^ sensitivity, we employed native thin filaments and myosin motors from cardiac samples from the same species and even from the same tissue sample.

### Isolation and purification of thin filaments and myosin from small cardiac tissue samples

**Figure 1:**
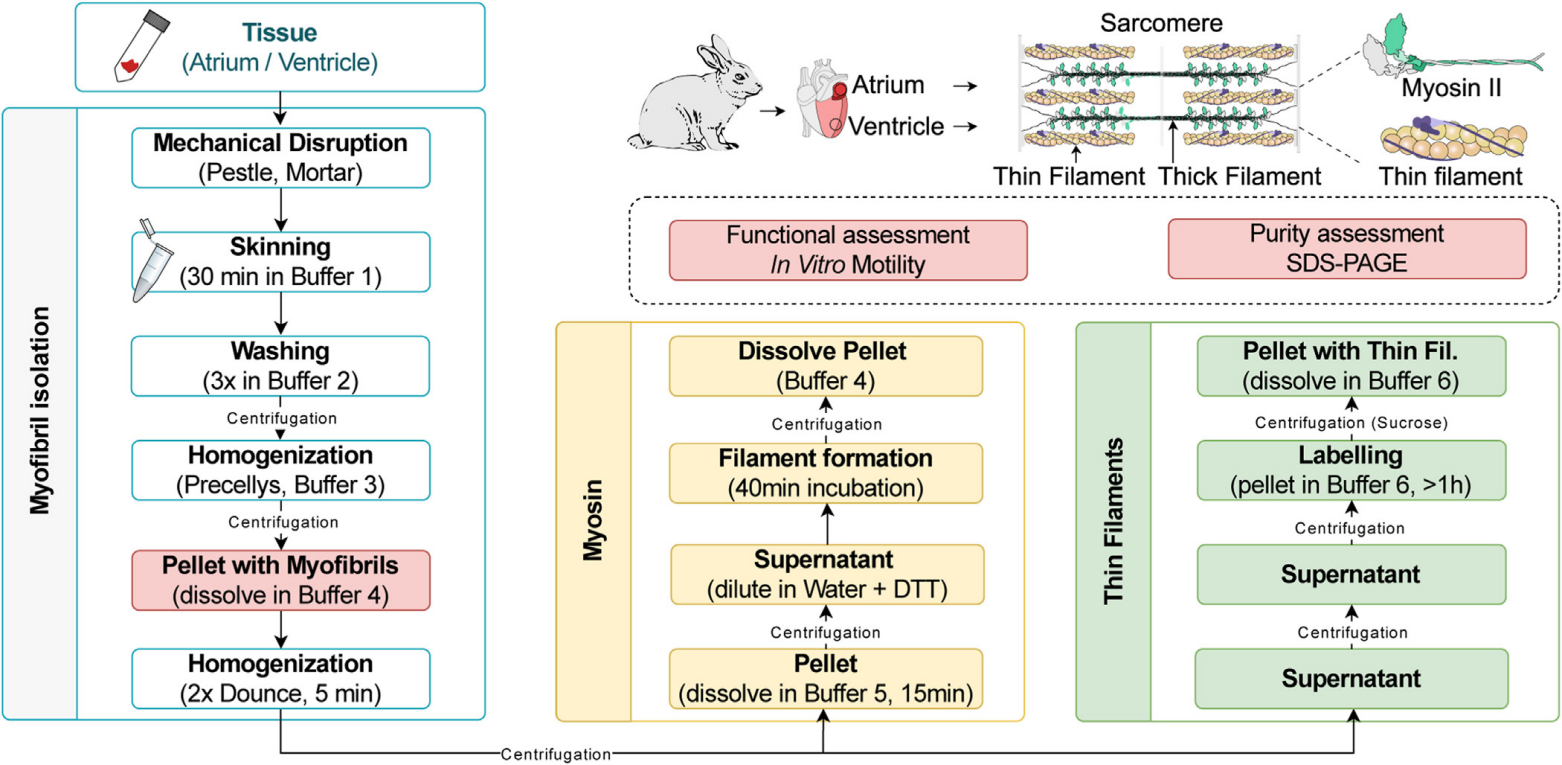
**Thin filament and myosin purification.** The diagram illustrates the workflow employed to extract and purify native thin filaments and myosin from the same small cardiac tissue sample.

The extraction of native thin filaments and myosin from small cardiac tissue samples follows a three-step approach (Fig. 1). The initial phase involves obtaining the myofibrils, followed by thin filament and myosin extraction. A schematic representation showing the key steps in the procedure is outlined in Figure 1.

Briefly, to obtain myofibrils, the tissue was mechanically disrupted into small pieces and collected into a tube containing 2,3-butanedione monoxime (BDM) and Triton X-100 in buffer 1. Subsequently, the pellets were washed using a solution without BDM and Triton X-100 (buffer 2) and gently dissolved in buffer 3 containing ATP. The initial homogenization was carried out using a Precellys system and myofibrils were collected from the homogenized samples by centrifugation. Pellets were resuspended in buffer 4 containing 10 mM MgATP, homogenized in a Dounce homogenizer, and then pelleted by centrifugation. At this step, the supernatant contained thin filaments, while the pellet contained myosin. Both samples were then processed in parallel, to purify thin filaments and myosin.

For thin filament purification, the supernatant from the previous step was initially cleared with a high-speed centrifugation, and the pellet containing the thin filaments was used to generate fluorescently marked thin filaments by adding Rhodamine-phalloidin. The protein preparation was further purified from contaminant proteins by running it through sucrose cushion buffer by centrifugation. Finally, the pellet containing native thin filaments was dissolved in the assay buffer (Buffer 6). The average yield of native thin filaments (as determined by Bradford assay) was 0.51 μg thin filaments per mg of initial tissue weight.

For the myosin extraction, the pellet was incubated for 15 min in buffer 5 containing high salt and ATP. This was followed by a centrifugation to remove the debris and a tenfold dilution in ultrapure water for 40 min to promote myosin filament formation. Myosin was pelleted by centrifugation and dissolved in buffer 5. The average yield of native myosin was determined to be about 1.75 μg per mg of tissue. The detailed protocol is further provided in experimental procedure section. The images of the key steps are given in Figure S1. Table S1 shows the thin filament and myosin yield of several individual preparations.

### Purity of isolated thin filaments and myosin

**Figure 2:**
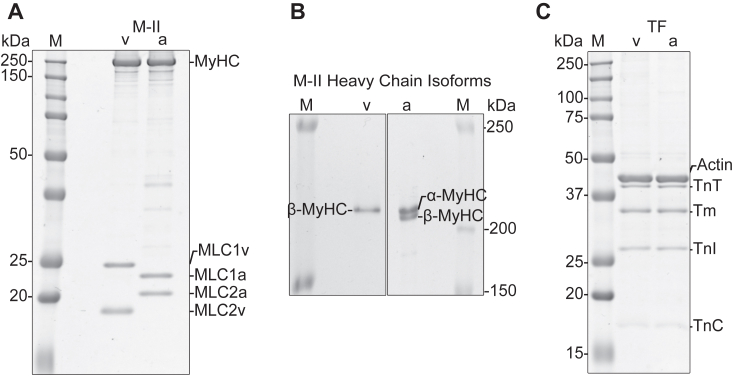
**Myosin and thin filament isolation using the newly established protocol.***A*, myosin extract (M-II) from the left ventricular (v) and left atrial appendage (a) tissue samples from rabbit, both associated with their characteristic light chains. First lane (M) is the protein ladder. *B*, myosin heavy chain (MyHC) isoform composition assessed under special SDS-PAGE conditions, showing the ventricular myosin with predominantly β-MyHC expression, while atrial samples showing double bands that correspond to α-MyHC and β-MyHC. The densitometric analysis using ImageJ yielded an average of ∼66% of α-MyHC and ∼34% of β-MyHC in atrial myosin preparations. *C*, native thin filaments (TF) extracted from the same tissue samples, containing all the components of the complex. The physiologic stoichiometry of 7:1:1 [actin: troponin (Tn): tropomyosin (Tm)] was approximately maintained.

The purity of native full-length myosin and thin filament samples obtained from rabbit ventricular (V) and atrial appendage (A) tissues using the above-mentioned protocol was assessed by SDS-PAGE. Figure 2*A* presents the myosin composition with the atrial- and ventricle-specific myosin heavy chains (MyHCs) and specific light chains, that is essential light chains (ventricular MLC1v and atrial MLC1a), and regulatory light chains (ventricular MLC2v and atrial MLC2a). To resolve the myosin heavy chain isoform composition, specific SDS-PAGE conditions were utilized (*cf.* M & M). As shown in Figure 2*B*, the ventricular tissue-derived myosin primarily exhibited a single band corresponding to the β-MyHC isoform (Source gel image in Fig. S2). Atrial tissue-derived myosin consistently contained a double band, suggesting the presence of a mixture of MyHC isoforms, that is, atrial-specific α-MyHC and β-MyHC. The densitometric analysis revealed that ventricular myosin samples predominantly expressed β-MyHC (without traces of α-MyHC detected). Note that less than 5% α-MyHC could not be detected in our isoform gels. Myosin extracted from atrial tissue consisted of ∼66% of α-MyHC and ∼34% of β-MyHC. The ratios of the two MyHC isoforms remained in this range for several independent preparations from the same and different animals. In Figure 2*C*, the protein composition of the native thin filaments extracted from the same tissue samples is shown, containing all the expected thin filament components, that is, actin, Tm, TnT, TnI, and TnC. The densitometric analysis showed a saturating concentration of regulatory proteins Tn and Tm. Figure S3 shows the source gel image of six different thin filament preparations, as well as the densitometric analysis.

### Functional analysis of cardiac tissue–purified native thin filaments and native myosin

**Figure 3:**
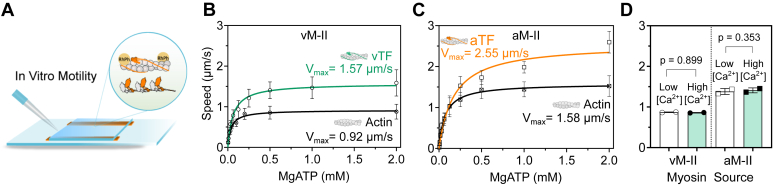
**Thin filament *versus* actin filament motility.***A*, illustration of *in vitro* motility assay using rhodamine-phalloidin–labeled thin filaments. *B*, ventricular thin filament and F-actin motility driven by ventricular myosins at different MgATP concentrations. The maximum actin filament gliding speed (V_max_) from the fit was 0.92 ± 0.03 μm/s (Mean ± SE) for F-actin and 1.57 ± 0.04 μm/s for native ventricular thin filaments (pCa 4.01). The ATP concentration to reach half-maximal velocity, that is, K_0.5_, was 38 μM and 63 μM, for F-actin and thin filaments, respectively (*p* = 0.027, extra-sum-of-squares F-test comparing the goodness-of-fit for K_0.5_ parameter). *C*, F-actin and atrial thin filament motility driven by atrial myosins at varied ATP concentrations. Atrial myosin exhibited a V_max_ of 1.58 ± 0.03 μm/s and a 2.55 ± 0.14 μm/s for F-actin and atrial thin filaments, respectively. The K_0.5_ was 72 μM and 174 μM, respectively (*p* = 0.003, extra-sum-of-squares F-test). Each data point in (*B*) and (*C*) represent the mean ± SD obtained from the Gaussian fit of the pooled mean filament speeds (101–340 manually tracked filaments), and the solid lines represent the hyperbolic fit to the Michaelis–Menten model in GraphPad Prism v.9.5.1. Thin filament gliding was measured at pCa 4.01. *D*, F-actin gliding driven by atrial and ventricular myosin at low and high [Ca^2+^]. The motility measurements were compared for low Ca^2+^ concentration in the motility buffer (pCa 7.57, ∼27 nM free Ca^2+^) and high calcium condition (pCa 4.01, ∼98 μM free Ca^2+^). The F-actin gliding speed for vM-II was 0.86 ± 0.15 μm/s and 0.86 ± 0.15 μm/s at low and at high [Ca^2+^], respectively (*p* = 0.899). For aM-II, the average speed was 1.43 ± 0.20 μm/s and 1.45 ± 0.17 μm/s at low and at high Ca^2+^, respectively (*p* = 0.353). Each data point represents a biological replicate (total 102–139 manually tracked filaments per condition). Statistical significance was determined by two-tailed unpaired *t* test.

The native proteins were employed in the *in vitro* TF motility assay to assess functional properties of the atrial and ventricular combinations (Fig. 3*A*). Motility assay allows examination of the ensemble molecule behavior of motor proteins as they interact with their respective cytoskeletal tracks under specific experimental conditions. Depending on their mechanical and enzymatic properties, the surface-immobilized myosin motors propelling actin filaments provide a useful assessment of actomyosin function. Factors influencing the chemomechanical properties of myosins could be associated with different filament gliding speeds.

The bare actin filaments (F-actin) can readily bind to myosin whereas the thin filaments comprising the regulatory tropomyosin–troponin (Tm–Tn) complex allow this binding primarily through Ca^2+^-induced conformational change along the native thin filaments making the binding sites on actin filament accessible to myosin. We first compared the F-actin and thin filament gliding in ATP concentration-dependent manner. F-actin gliding over ventricular myosin II–coated surface was performed at varying ATP concentrations from 4 μM to 2 mM MgATP. The moving actin filaments were tracked for their gliding speed. Average speeds recorded at different ATP concentrations exhibited an asymptotic trend. Data was fitted using the Michaelis–Menten equation, resulting in F-actin maximum speed V_max_ of 0.92 ± 0.03 μm/s (Mean ± SE). Subsequently, thin filament gliding was assessed by employing native thin filaments from the same source of tissue from which the myosin was extracted. These experiments were carried out at varying ATP concentrations and at saturating calcium concentrations of pCa 4.01 (pCa = −log[Ca^2+^]). As shown in Figure 3*B*, ventricular thin filaments moved at a maximum speed of 1.57 ± 0.04 μm/s. The ventricular myosin exhibited a slower F-actin gliding speed than myosin derived from the atrium (Fig. 3*C*), that is, maximum speed of 0.92 μm/s for ventricular myosin II and 1.58 ± 0.03 μm/s for atrial myosin II. Similarly, the atrial thin filaments propelled by atrial myosins displayed higher maximum speed, that is, 2.55 ± 0.14 μm/s, than both F-actin– and ventricular myosin–driven ventricular thin filaments. Thus, the regulatory Tm–Tn complex potentiated the gliding speed by about 1.7 fold in proteins derived from both the ventricle (Fig. 3*B*) and atrium (Fig. 3*C*). Besides, the K_0.5_ value, indicating the ATP concentration required to achieve half-maximal speed, was ∼1.7 fold higher for thin filament than F-actin for ventricular myosin (K_0.5_ using F-actin was 38 μM, and thin filaments was 63 μM). For atrial myosin, the ratio was ∼2.4 fold (F-actin K_0.5_ = 72 μM, thin filament K_0.5_ = 174 μM). We further probed if the observed increase in the thin filament gliding speed is a result of the effect of Ca^2+^ on myosin’s enzymatic properties. As shown in Figure 3*D*, the addition of Ca^2+^ in the motility buffer did not significantly improve the F-actin gliding speed propelled by either atrial or ventricular myosin. For ventricular myosin, F-actin gliding speed remained constant at about 0.9 μm/s in the absence and presence of Ca^2+^. Similarly, for the atrial myosin, the speed was about 1.4 μm/s with and without Ca^2+^. Thus, the observed influence on the gliding speed of thin filaments can be attributed to the Tm–Tn complex. Apart from revealing important differences between atrium- and ventricle-derived proteins, these experiments ensured that gliding speed was solely constrained by the ATP concentration and not influenced by factors such as insufficient availability of functional myosin motors on the surface.

### Dependence of thin filament gliding speed on Ca^2+^ concentration

**Figure 4:**
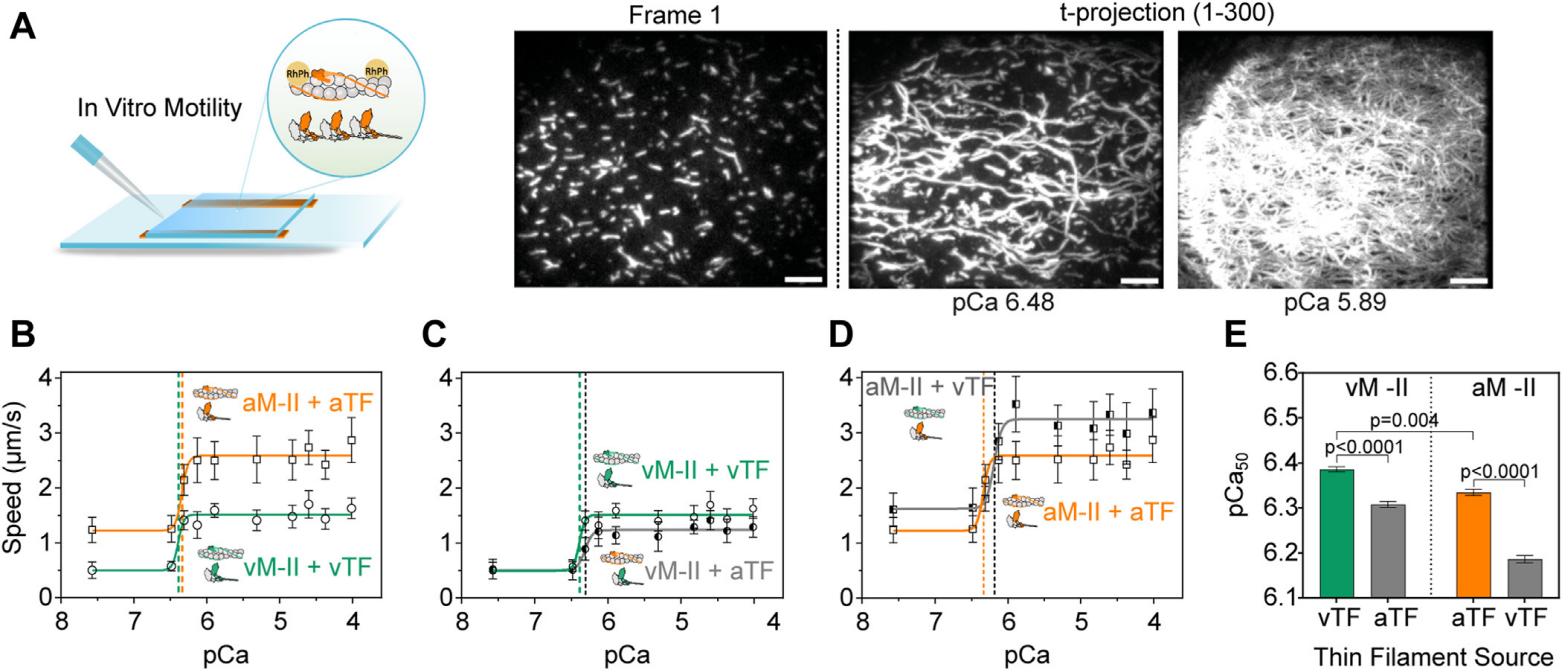
**Speed-pCa analysis.***A*, an image (frame 1) showing a typical distribution of thin filaments in the motility chamber. The t-projection (average TF position over time) generated from the tiff-stack visualizes the extent of TF movements at a low Ca^2+^ concentration of 332 nM (pCa 6.48) and a higher Ca^2+^ concentration of 1.3 μM (pCa 5.89), in a motility chamber with native vM-II and vTF extracted from the same tissue sample. Scale bars represent 5 μm. *B*, myosin and native thin filaments extracted from the same atrial or ventricular tissue samples were analyzed under different calcium concentrations. The data points were fitted with the Hill equation to estimate the maximum velocity and pCa_50_. At saturating calcium concentrations, the movement driven by ventricular myosin shows a maximum speed of 1.51 ± 0.01 μm/s (Mean ± SE) and by atrial myosin of 2.58 ± 0.01 μm/s. The calcium sensitivity considered from the pCa_50_ (calcium concentration to achieve half-maximal gliding speed) for ventricle-derived myosin and thin filaments was 6.385 ± 0.006 (Mean ± SE), and atrium-derived proteins was 6.334 ± 0.007. *C*, speed-pCa analysis with swapped thin filaments. Atrial thin filament motility on the surface coated with ventricular myosin decreased the maximal gliding speed from 1.51 ± 0.01 μm/s to 1.24 ± 0.01 μm/s. *D*, the ventricular thin filament gliding speed on the surface coated with atrial myosin was higher (3.25 ± 0.02 μm/s) than that of atrial thin filaments (2.58 ± 0.01 μm/s). Data points in (*C*) and (*D*) are mean speed ± SD values obtained from the Gaussian fit of 201 to 307 manually tracked filaments from three preparations (in swapping experiments 101–209 filaments from two preparations). The pCa *versus* speed response curve with variable slope was fitted in GraphPad Prism v.9.5.1. *E*, the bar graph with pCa_50_ ± SE values, resulting from the nonlinear fits of data in (*C*) and (*D*), shows the change in pCa_50_ when atrial and ventricular thin filament motility was examined in combination with different myosins. The *p*-values from the extra-sum-of-squares F-test assessing the goodness-of-fit for this parameter were indicated in the bar graph.

After comparing the fundamental speed differences for actin and thin filament gliding over atrial and ventricular myosins, the calcium sensitivity of thin filaments was characterized in motility experiments conducted under varying calcium concentrations. The motility buffer contained 2 mM ATP and free Ca^2+^ concentrations ranging between a minimum of 27.0 nM to a maximum of 97.8 μM corresponding to pCa of 7.57 and 4.01, respectively. While there were fewer thin filaments moving slowly on the myosin-coated surface at low Ca^2+^, the number of moving filaments and speed of movement increased at higher Ca^2+^ (Movie S1 and t-projections in Fig. 4*A*). Plotting gliding speed over pCa resulted in an s-shaped curve reminiscent of force-pCa curves obtained in muscle fiber studies. The steep rise in the velocities within a short pCa range was typically observed for the speed-pCa plot. Thus, speed increased over increasing Ca^2+^ concentrations and reached a plateau (Fig. 4*B*). The resulting datasets exhibiting a sigmoidal curve were fitted using the Hill equation, that is, dose-response stimulation model in GraphPad Prism (log(agonist) *versus* response with variable slope). The fit parameter logEC_50_ or pCa_50_ represents the calcium concentration necessary to achieve half-maximal gliding speed and was used to assess the calcium sensitivity of motility. Notably, proteins derived from both the atrium and ventricle exhibited small differences in pCa_50_ values (Fig. 4*B*), indicating ventricular TFs to be relatively more Ca^2+^-sensitive. Specifically, pCa_50_ for ventricle-derived myosin and thin filaments was 6.385 ± 0.006 and for atrium-derived proteins was 6.334 ± 0.007 (Mean ± SE). The extra-sum-of-squares F test for the goodness-of-fit showed that the datasets were different for this parameter (*p* < 0.004, depicted in Fig. 4*E*). Furthermore, the maximal thin filament gliding speed at saturating calcium concentrations, as indicated by the top values of the fits, was similar to the V_max_ obtained from the previous experiments for ATP dependence of motility (Fig. 3). For ventricular myosin-driven motility, the maximal speed was 1.51 μm/s and for atrial myosin-driven motility was 2.58 μm/s, when native thin filaments from the same tissue were employed.

### Effect of thin filaments on the myosin-driven gliding speed

**Figure 5:**
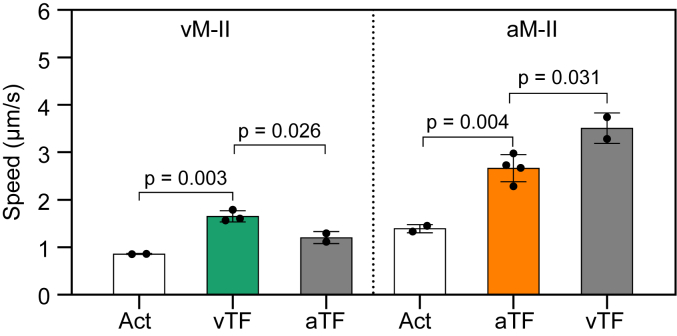
**Effect of filament source on motility speed.** The average gliding speeds of F-actin (Act), tissue-specific, and swapped thin filaments propelled by ventricular and atrial myosin are compared. The experiments were performed in the presence of 2 mM MgATP and pCa 4.01. Each data point represents one biological replicate (with a total of 102–214 filaments manually tracked filaments per condition). *p*-values (mean ± SD) for pairwise comparisons were calculated using two-tailed unpaired *t* test in Prism 9.5.1.

To evaluate the potential influence originating from the source of Tm–Tn on the gliding speed, swapping experiments were conducted. Here, surfaces coated with ventricular myosin were assayed using labeled atrial thin filaments and *vice versa*. Surprisingly, there was significant variation in maximal gliding speed and calcium sensitivity observed upon swapping. Specifically, ventricular myosin–driven atrial thin filament movements had a decreased maximum speed of 1.24 μm/s compared to 1.51 μm/s for the native ventricular combination (Fig. 4*C*). Conversely, the atrial myosin-driven ventricular thin filament had a higher maximum speed of 3.25 μm/s than the native atrial combination (2.58 μm/s) (Fig. 4*D*). Furthermore, calcium sensitivity exhibited a consistent decrease in both non-native combinations. The ventricular myosin in combination with atrial thin filaments decreased the pCa_50_ to 6.308 ± 0.007 (Mean ± SE), compared to 6.385 ± 0.006 with ventricular thin filaments. Similarly, the use of ventricular thin filaments on atrial myosin-coated surface decreased the pCa_50_ from 6.334 ± 0.007 to 6.186 ± 0.008 (Fig. 4*E*). These observations showed thin filament source-specific differences resulting in significant functional consequences, that is, nearly 20% decrease or increase in the gliding speed (summarized in Fig. 5) and change in Ca^2+^ sensitivity.

### Phosphorylation levels of thin filament components

**Figure 6:**
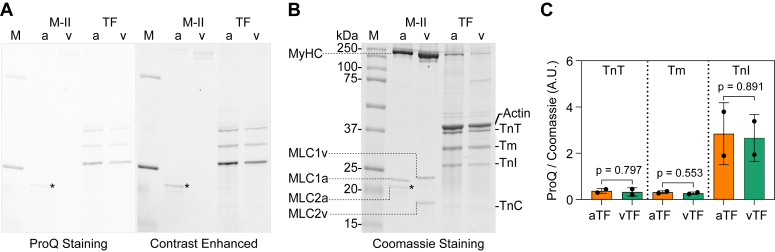
**Phosphorylation levels of proteins in myosin and thin filament samples obtained from atrium and ventricle.***A*, ProQ Diamond stained SDS-PAGE gel depicting protein phosphorylation. The *left* image shows the original and the *right* one a contrast-enhanced image of the gel. Phosphorylated forms of TnT, Tm, TnI, and MLC2a (shown with an *asterisk*) are discernible. *B*, corresponding Coomassie stained gel shows the total protein content. *C*, densitometric analysis using the ProQ/Coomassie ratio reveals no significant differences in the phosphorylation levels of aTF and vTF proteins TnT, Tm, and TnI in two different preparations. The statistical significance was checked using the two-tailed unpaired *t* test.

One possible source of the observed differences in the thin filament motility could be PTMs of the Tm–Tn complex. Therefore, we examined phosphorylation of thin filament components. Freshly isolated atrial and ventricular protein samples prepared in parallel were run on the gel and stained to detect the phosphorylation status of corresponding proteins. As shown in Figure 6, *A*–*C*, no significant difference was observed in the phospho-modified levels of Tm, TnT, and TnI between atrial and ventricular thin filaments. These results suggest the involvement of factors other than phosphorylation to be responsible for the detected disparity, when either atrial or ventricular nTFs were used.

## Discussion

Among the main strengths of this study is the improved protocol to isolate myosin and native thin filaments from the same tissue source, which proved successful even for myectomy-sized tissue samples.

Primary observations from studying the native thin filaments include the following:

(1) The positive influence of ventricular tissue–derived thin filaments on the atrial myosin-driven gliding speed.
(2) The negative impact of atrial thin filaments on ventricular myosin-propelled gliding speed.
(3) The difference in calcium sensitivity as derived from the pCa_50_ depending on the source of thin filaments.
(4) Phosphorylation of the individual nTF components do not appear to be a factor responsible for the observed effects of thin filament source under our experimental condition.

### Advantage of a new protocol

In our newly established protocol, the amount of tissue necessary to prepare the thin filaments can be as small as 10 mg. This robust protocol works with tissue samples ranging from 10 mg up to 250 mg. The other important feature of the protocol is a streamlined, efficient procedure that yields pure thin filaments from frozen tissue in a day, what in earlier standard procedures required a week.

In conventional preparations of thin filaments, a starting material of >150 g (28) was used. The reduced tissue amount used in the past was 2 g to isolate troponin (29) and 100 g minced muscle to isolate Tm–Tn complex (30, 31). At least two reports have indicated the starting tissue amount of 30 mg to prepare native thin filaments to use in electron microscopy studies (32, 33). Bacterially purified thin filament components followed by reconstitution is one of the widely employed methods to generate the reconstituted TFs, which yet again require nearly 1 to 2 weeks time from plasmid transformation to the final reconstitution (34). Apart from downsizing of the starting material (to 10 mg), the extraction of myosin motors and corresponding native thin filaments from the same tissue sample is another major advantage to study the endogenous combination of thin filament-myosin. This has created a possibility to probe the patient cardiac or skeletal muscle tissue for disease-specific alterations in the contractile function. The advanced protocol was successfully tested for mouse and human cardiac tissue to purify nTF and myosin (data not shown). Overall, the procedure has the advantage of reduced source material and improved and purer final yield that can be extended to muscle tissue from diverse species while reducing the time required for preparation.

### Native TFs *versus* reconstituted TFs

On average, native thin filaments were about 1 μm long. Presence of some longer than 1 μm TF suggested that the capping proteins may have been lost during the purification procedure and the thin filaments fused end-to-end, forming longer filaments. When the TF solutions were kept for more than 2 days at 4 °C, the regulation *via* Ca^2+^ gradually diminished over time, suggesting that the regulatory components dissociated from the actin filaments, thus requiring no activation through Ca^2+^. Therefore, we constrained the use of TFs to two days after resuspending and labeling of the TFs.

Reconstituted TFs are claimed to be the optimal alternative to nTFs to examine their Ca^2+^-dependent activation. However, the nTFs bear an advantage by preserving the PTMs that might be very relevant for the TF function. The mismatched protein partners or individual subunits may result in subtle yet critical disruptions in the calcium-mediated thin filament and myosin interactions. A comprehensive understanding of the observed functional data is possible by employing native protein sources that ensure the correct composition and closely reflect the intricacies of the complex interactions, mimicking the *in vivo* setup.

### Thin filament motility

We observed that the unregulated or bare actin filament moved with a slower velocity than the nTF by about 1.7 fold for both atrial and ventricular myosin. This observation is consistent with previous reports (35, 36). The difference in the actin filament gliding speed driven by atrial *versus* ventricular myosin was also reported earlier for pig cardiac myosin, although the measured speed were higher than our measurements for rabbit cardiac myosins (37). The likely reason for this disparity is their experimental conditions where the motility measurements were performed at 32 °C. Note that the myosins were assessed for their mechanical activity at 22 °C in our current study. One might assume that the readily available actin-binding sites for myosin in unregulated actin filaments will allow the optimal actin filament gliding because the myosin cross-bridge recruitment would not be a limiting factor as in the thin filaments. The gliding speed is primarily determined by the actomyosin detachment rate and the powerstroke size. The difference in speed may result from the possibility that the regulatory components, that is, the Tm–Tn complex, influence the rate of cross-bridge cycling by changing the duration of the strongly-bound actomyosin states and, thereby, the actomyosin detachment rate. The question is which steps of the ATPase cycle are influenced by Ca^2+^ and to what extent. Within the ATPase cycle, Pi and or ADP release rates are expected to influence the actomyosin detachment rate. In agreement with this, both Pi and ADP release rates were found to be influenced by the presence of Tm–Tn in the thin filaments activated by Ca^2+^. In solution studies, Ca^2+^-mediated activation of thin filaments resulted in a 47-fold increase in Pi release as measured using phosphate-binding protein when compared with the absence of Ca^2+^ (25). Besides, the ADP release was assumed to be the likely rate-limiting step for thin filament motility at maximal calcium activation (38). In solution kinetics measurements, the rate of ADP release from regulated actomyosin was 15-fold greater in the presence of calcium (39), suggesting that the actin filament-bound regulatory proteins can modulate ADP release from myosin in a Ca^2+^-dependent manner.

Another interesting observation was the increased thin filament velocity as a function of Ca^2+^. Rather than exhibiting an on-off switch, that is, nonmotile to the motile thin filament, others and we observed a graded increase in the velocity. Earlier reports have implied different mechanisms to support the graded activation, for example, direct Ca^2+^ control of cross-bridge cycling (40), change in the cross-bridge duty cycle (41, 42), the load on the filaments limiting the speed (43, 44), or enhanced cross-bridge recruitment, that is, Ca^2+^ concentration-dependent myosin binding to actin (45, 46).

The direct effect of Ca^2+^ in controlling the myosin ATPase cycle seems unlikely, as we did not observe an effect of Ca^2+^ on the bare actin filament gliding speed (Fig. 3*D*). An indirect effect on cross-bridge cycling *via* Ca^2+^ regulation of thin filaments, changing the duration of force-generating ADP.Pi and ADP-bound actomyosin states (or detachment rate) seems likely. A change in the cross-bridge duty cycle is expected if the lifetime of the strongly bound ADP.Pi and ADP-bound actomyosin state is altered, provided the overall ATP turnover rate remains constant. We observed that even in the absence of or very low, Ca^2+^ concentration thin filaments immobilize on the myosin-coated surface. Two possible explanations for this finding are: (1) multiple weak interactions between myosin and actin complex keeping the filament attached to the myosin coated surface, that is, the presence of Tm–Tn do not hinder these interactions, (2) few sites along the thin filaments lost the regulatory components, thereby permitting the Ca^2+^-independent interactions. The first possibility of weak interactions implies that in the absence of Ca^2+^ and at low Ca^2+^ concentrations, weakly bound cross-bridges contribute to a drag force, leading to slower TF gliding speed. This notion was also considered in an earlier report (46). Accordingly, low Ca^2+^ supporting fewer active cross-bridges involved in pulling on the thin filaments are slowed down by multiple weakly bound cross-bridges creating a drag force, retarding force-generating cross-bridges. Thus, the concentration of Ca^2+^ is negatively correlated to the number of weak interactions. An increase in [Ca^2+^] shifts the equilibrium towards the number of force-generating cross-bridges and, thereby, reduces the number of drag-generating cross-bridges. The corresponding velocities are probably the result of a calcium-dependent modulation of rate-limiting steps in the cross-bridge cycle for gliding.

Our observations, in combination with earlier studies, suggest that the improved recruitment of force-generating cross-bridges with increasing Ca^2+^ and the rather indirect effect of Ca^2+^ *via* modulation of the kinetics of cross-bridge cycling may not be mutually exclusive. Both processes may contribute to the regulation of thin filament gliding speed through these mechanisms. This argument is further strengthened by the observation that the Ca^2+^ do not directly affect the myosin ATPase cycle (Fig. 3*D*), meaning the mediation is through actin regulatory components Tm–Tn.

### Atrial and ventricular thin filament subunit isoforms and PTMs

The observed differences in the gliding speeds of atrial and ventricular thin filaments are noteworthy as there are no reports of major isoform differences described for Tm and Tn in rabbit heart chambers. In rabbits, only the α-isoform of Tm is expressed in the heart, while in the human heart, α- and β- Tm isoforms are expressed at a 4.8:1 ratio (47). A single TnC isoform is reported in heart muscle (cTnC) (48). In the rat and adult human heart, only one cTnI isoform is found (49). At least four isoforms of cTnT (cTnT1, cTnT2, cTnT3, and cTnT4) are expressed in a developmentally regulated manner in the human heart (50). Abnormal cTnT isoform expression has been linked to heart disease (51). Alpha-tropomyosin and cardiac troponin T mutations cause hypertrophic cardiomyopathy (a disease of the sarcomere). Age-dependent changes in the cTnT isoforms have also been reported in rabbit hearts (52).

PTMs such as phosphorylation of the regulatory subunits can change the functional output of the sarcomere (53). TnT and Tm can also be phospho-modified. However, in our phosphoprotein gels, we did not observe any obvious discrepancy between the atrial and ventricular TF component phosphorylation levels (Fig. 6). Further detailed mass spectrometry analysis is expected to reveal the responsible factors contributing to the TF-specific effects. However, this is outside the scope of the current manuscript and would be pursued further in a future goal. The animals used in our studies for multiple experiments are approximately the same age, and atrial and ventricular tissue from the same animal is compared. So, it is unlikely that the observed effect arose from an age-related isoform difference.

### Conclusion

Overall, we have developed a streamlined and rapid procedure to isolate native thin filaments and myosin from cardiac muscle. The physiological stoichiometry of the regulatory components in the thin filaments and native interaction partners, that is, myosins, is an important attribute of such an approach, which can be widely applied to other muscle types. We provide evidence that not only the myosin isoform but also the thin filaments are important for the mechanical function of the system. Apart from gaining fundamental insights into the tissue-specific composition of myosins and thin filaments for heart chamber function, these investigations are directly relevant to myopathy studies. In heart failure and cardiomyopathies, TF regulatory components isoform switching and changed PTMs of some of the subunits are reported. The approach in the current study can be particularly useful in examining the properties of the correct endogenous TF-myosin combinations, permitting the usage of small donor or patient myectomies to unravel the pathomechanism responsible for cardiac dysfunction.

## Experimental procedures

### Animal tissues

The cardiac tissues were collected from New Zealand white rabbits, Crl:KBL (NZW). The animals were euthanized as per the guidelines from German animal protection act §7 (sacrifice for scientific purposes). In this study, we used shared organs originating from the animals approved for experiments with authorization number 18A255. The animals registered under reference number G43290 were obtained from Charles River France. All the procedures were carried out in accordance with relevant guidelines and regulations from the Lower Saxony State Office for Consumer Protection and Food Safety and Hannover Medical School, Germany.

Left atrial appendage and left ventricular wall samples were collected from the rabbits 10 to 20 min post-mortem and were immediately immersed in the ice cubes of Custodiol solution. Subsequently, the tissues were cut into smaller pieces and promptly snap-frozen in liquid nitrogen.

### Thin filament and myosin extraction

Our purification protocol was optimized to obtain native thin filaments and myosin from the same small-sized tissue of ∼10 to 250 mg in a day, giving the opportunity to assess the function of the proteins in their native composition for a relatively short time.

Note that the following detailed protocol and the corresponding buffer volumes are provided for about 100 mg of starting tissue weight. Note that we were able to downscale the amount to 10 mg of starting tissue, yielding sufficient quantities of functional proteins for motility experiments. The extraction of proteins from the tissues follows a three-step approach. The initial phase involves obtaining the myofibrils, followed by thin filament and myosin extraction (outlined in Fig. 1). To obtain myofibrils, the tissue was mechanically disrupted into small pieces in liquid nitrogen, using a pestle and mortar, followed by the collection of the pieces into a 1.5 ml tube containing 1 ml of pre-chilled skinning solution or buffer 1. Buffer 1 had a pH of 7.4 and contained 3 mM Na_2_ATP, 5 mM Mg-acetate, 5 mM ethylene glycol-bis (2-aminoethylether)-N,N,N′,N′-tetraacetic acid (EGTA) (Sigma-Aldrich, E4378), 50 mM creatine phosphate (Sigma-Aldrich 2380), 5 mM KH_2_PO_4_, 8 mM NaN_3_, supplemented with fresh 1 mM DTT (Sigma-Aldrich, D0632-10G), 1× cOmplete EDTA-free Protease Inhibitor Cocktail (Roche, 05056489001), 20 mM BDM (Sigma, B0753), and 1% Triton X-100 (Roche, 11332481001). The samples were incubated for 30 min on ice with intermittent gentle mixing using a plastic spatula, followed by the removal of buffer 1 through centrifugation at 300*g* for 2 min. Subsequently, the pellets were washed twice with 1 ml of buffer 2 (same as buffer 1, but without BDM and Triton X-100) without incubation in the buffer, followed by a third wash including a 15 min incubation in buffer 2. Buffer 2 was removed by centrifugation at 300*g* for 2 min during the first two washes and at 400*g* for 5 min at the last washing step. Subsequently, the washed pellet was gently dissolved in 350 μl of buffer 3. Buffer 3 had a pH of 7.0 and contained 10 mM imidazole, 25 mM propionic acid (Sigma-Aldrich, P1386), 10 mM caffeine (Sigma-Aldrich, C0750), 10 mM creatine phosphate, 2 mM MgCl_2_, 3 mM EGTA, 2 mM MgATP, and freshly added 2 mM DTT and 1 mM 4-(2-Aminoethyl)benzene-1-sulfonyl fluoride (AEBSF) Hydrochloride (AppliChem, A1421). The initial homogenization was carried out using the Precellys system (Bertin, Precellys 24), employing 0.5 ml tubes with ceramic beads inside (two 2.8 mm beads and five 1.4 mm beads per tube). Six tubes were used per sample in a run of 10 s at 5000 rpm. The resultant myofibrils were collected from the homogenized samples by centrifugation (400*g* for 10 min). Pellets were resuspended in a total volume of 400 μl of buffer 4 (same as buffer 3 except having 10 mM MgATP and 10 mM DTT) and homogenized in 1 ml Dounce homogenizer for 5 min on ice. The resulting samples were then centrifuged at 2400*g* for 7 min. The supernatant was collected in an ultracentrifuge tube and the pellet was re-suspended in 400 μl total volume of buffer 4. Following another 5 min homogenization in Dounce homogenizer, samples were pelleted again, by centrifugation at 2400*g* for 15 min. The supernatant was collected in the same ultracentrifuge tube. At this step (so-called ‘*critical step*’), the supernatant contained thin filaments, while the pellet comprised myosin. Both pellet and supernatant were processed in parallel to further purify the myosins and thin filaments as explained below.

For thin filament purification, the supernatant from the previous step was initially cleared with a high-speed centrifugation for 10 min at 45,000*g*. The resulting supernatant was divided into two tubes and centrifuged again in TLA 120.2 rotor at 150,000*g* for 1:45 h to pellet the cleared thin filaments. These thin filament pellets can be stored for two weeks on ice, without compromising their functionality. Before use, the pellet is overlaid with 30 μl of buffer 6 (motility assay buffer) containing 25 mM imidazole hydrochloride at pH 7.2, 25 mM NaCl, 4 mM MgCl_2_, 1 mM EGTA, supplemented freshly with 2 mM DTT and 1 mM AEBSF. Following a 30 min incubation, the pellets were gently dissolved by pipetting. Rhodamine-phalloidin (Sigma-Aldrich; P1951) of 0.25 nM was added to label the filaments overnight on ice (or, minimum for 1 h). Next, the thin filaments were further purified and the contaminant proteins were removed by overlaying the solution on sucrose cushion buffer (same composition as buffer 6 with additional 20% sucrose) and by centrifugation in TLA 120.2 rotor at 150,000*g* for 1:45 h. The final pellet was overlaid with 30 μl buffer 6 to resuspend the thin filaments in the pellet for ∼30 min and then gently dissolved by pipetting. The average yield of native thin filaments as determined by Bradford assay was about 0.51 μg thin filaments per mg of initial tissue. The thin filaments were tested for their composition and stoichiometry in SDS-PAGE gel and employed in the thin filament gliding assay to assess their regulation.

For the extraction of myosin, the pellet from the ‘*critical step*’ was incubated for 15 min in buffer 5, which contained 500 mM NaCl, 10 mM 4-(2-hydroxyethyl)piperazin-1-ethanesulfonic acid pH 7.0, 5 mM MgCl_2_, 2.5 mM MgATP, supplemented with fresh 2 mM DTT and 1 mM AEBSF. This was followed by a centrifugation step using TLA 120.2 rotor at 150,000*g* for 45 min to remove the debris. The resulting supernatant was diluted tenfold in ultrapure water containing 2 mM DTT and incubated for 40 min on ice, with intermittent gentle mixing to promote myosin filament formation. Myosin was pelleted by centrifugation in TLA 110 rotor at 70,000*g* for 45 min and dissolved in an appropriate volume of buffer 5, aiming to achieve a concentration above >3 mg/ml of protein. The average yield of native myosin was determined to be 1.75 μg per mg of starting tissue.

Throughout the extraction process, all tubes and pipette tips were low protein binding, all centrifugations were done at 4 °C, all incubation and homogenization steps were done on ice, and care was taken to pre-chill the solutions and tubes on ice before use. For low-speed (<2500*g*) centrifugations in this protocol, Heraeus 7599 rotor was used (Thermo Fisher Scientific, Sorvall Primo R Benchtop). For ultra-centrifugations, TLA 120.2 or TLA 110 rotors were used (Beckman Coulter, Optima).

### Actin filament preparation

G-actin was prepared from chicken pectoralis muscle following the protocol outlined in the referenced publication (54). For *in vitro* actin filament motility assays, bare actin filaments (F-actin) were generated by incubating G-actin in polymerization buffer (p-buffer) containing 5 mM Na-phosphate, 50 mM K-acetate, and 2 mM Mg-acetate, supplemented with 0.5 mM AEBSF protease inhibitor overnight at 4 °C. An equimolar concentration of fluorescent phalloidin (tetramethylrhodamine) (Sigma-Aldrich; P1951) was added to fluorescently mark the actin filaments. Unlabeled actin filaments were prepared the same way except for the addition of phalloidin. A control experiment showed no difference in actin gliding speed between actin prepared from chicken and rabbit fast skeletal muscles (Fig. S4).

### *In vitro* motility assay

Motility assay was carried out as described earlier (55), with minor changes. Briefly, glass slides were cleaned two times with ultrapure water and one time with HPLC water in a sonicator for 15 min each and dried for 8 h at 55 °C. Flow chambers were prepared using double-sided tape (TESA), to result in ∼5 μl chamber volume. After 5 min of incubation with buffer 6 containing 0.5 mg/ml bovine serum albumin (Sigma, A-6003), full-length native myosin with ∼1 mg/ml concentration was injected into the chamber and allowed to bind for 5 min. This was followed by the washing of unbound myosin using buffer 6 with bovine serum albumin. The nonfunctional myosin motors were blocked by using 0.5 μM unlabeled actin for 1 min, followed by extensive washing using buffer 6 containing 2 mM ATP, to release the active myosin heads. The chamber was washed with buffer 6 and labeled thin filaments were injected for 1 min. The final motility buffer contained buffer 6 with 2 mM ATP and the anti-bleaching system (containing a final of 10 mM DTT, 18 μg/ml catalase, 0.08 mg/ml glucose oxidase, 10 mg/ml D-Glucose), together with the appropriate amount of calcium depending on the desired free calcium. Free calcium concentrations in the presence of 1 mM EGTA and 2 mM ATP metal chelators were calculated using the recently published web application for metal chelator calculations, PyChelator (56), with the following parameters: pH 7.2, ionic equivalence of 40 mM, 22 °C chamber temperature, and 6 mM Mg^2+^. The constants from NIST database #46 v8 were used (https://www.nist.gov/srd/nist46, December 15, 2023). Control experiments were done to rule out any possible impact of 4 mM ionic strength increase on motility upon the addition of calcium (Fig. S5).

### Total internal reflectance fluorescence microscopy

The assay chambers were placed on an inverted microscope in our custom-made four-channel simultaneous-imaging TIRF setup described in detail previously (55). The Rhodamine-phalloidin–labeled filaments were excited using a 532 nm Nd:YAG laser (Coherent, Compass 315M-150 SL) and recorded using an Andor camera (Andor Technology, iXon DV887). The stage temperature was kept constant at ∼22 °C. Images were acquired with a time resolution of 0.2 s (*i.e.*, 5 frames/sec). Movies of 300 frames were generated using the Andor Solis software, resulting in 256 × 256 pixel tiff-stacks with 170 nm/pixel resolution and 32 bits/pix.

### SDS-PAGE analysis

The protein composition of myosin and thin filaments was assessed using 12.5% SDS-PAGE, in a mini-Protean Tetra system from Bio-Rad. The acrylamide/bisacrylamide ratio was set to be 37.5:1 using RotiphoreseGel 30 (Roth, 3029.1). Samples were loaded at ∼4 μg per lane. Coomassie staining was done for 30 min using Quick Coomassie Stain (Biotrend, NB-45-00078-1L). 16 bit tiff images were taken in ImageQuant LAS 4000 system, at 2 s exposure time.

To resolve the MyHC isoforms from rabbit cardiac samples, SDS-PAGE was performed under specific conditions. The stacking gel was composed of 5% glycerol and 5% acrylamide/bisacrylamide (100:1, using RotiphoreseGel 30 and RotiphoreseGel A from Roth 3029.1 and 3037.1, respectively) (57). The resolving gel had 6.5% AA/BisAA with 10% glycerol. Gels were run in the CBS Vertical System from CBS Scientific (20 cm total length, 4 cm allocated for stacking gel, and 16 cm for the resolving gel). Myosin samples were loaded to have 0.25 μg per lane and the gel was run at room temperature for 21 h employing a constant current of 8 mA in the first 1.5 h followed by 10 mA for the rest of the time (with max. 150 V and 20 W).

### Phosphoprotein gels

The protein phosphorylation levels of the thin filaments and myosins were assessed as described before (5). The pellets of myosin and thin filaments were dissolved in 1D buffer, composed of 62.5 mM Tris, pH 6.8, 15% glycerol, 1% SDS, and 0.002% bromophenol blue, supplemented freshly with one tablet of PhosSTOP (Roche; 4906837001) per 1 ml of 1D buffer. 12% Criterion TGX Precast Midi Protein Gels (Bio-Rad, 5671043) were used to resolve the proteins loaded as 3 μg for thin filaments and 4 μg for myosin. The gel was stained with ProQ Diamond (Molecular Probes, P33301) and imaged with 45 s exposure time (GE Healthcare, ImageQuant LAS 4000). Subsequently, to compare the phosphorylation levels with the total protein, the gel was stained with Coomassie stain and imaged with 2 s of exposure time. ProQ/Coomassie ratio was used to compare the groups.

### Data analysis and statistics

All densitometric analysis of gels was performed using ImageJ’s built-in gel analysis tool (See Fig. S3 for thin filament densitometric analysis). The signal intensities of individual bands and their relative proportion were additionally confirmed by the deconvolution of signals using OriginLab Software (OriginLab Corporation).

F-actin and thin filament gliding data from *in vitro* motility studies were analyzed by manual tracking of the smoothly moving filaments employing the MTrackJ plugin v3.0.0 (58) in Fiji v1.54g (59). The mean ± SD for each condition was obtained from the Gaussian fit of the pooled mean filament speeds. GraphPad Prism v.9.5.1 was used to analyze the data. Further details of filament and biological preparation numbers, together with the statistical tests used, are given in the respective figure legends.

## Data availability

All data supporting the findings of this study are contained within the article and as supplemental information.

## Supporting information

This article contains supporting information.

## Conflict of interest

The authors declare that they have no conflicts of interests with the contents of this article.
