# Supplementary material for: Influence of native thin filament type on the regulation of atrial and ventricular myosin motor activity
Source: J Biol Chem. 2024 Oct 5;300(11):107854. doi: 10.1016/j.jbc.2024.107854 (PMC11570844; doi:10.1016/j.jbc.2024.107854)
Supplement: Supporting Information [file mmc1.pdf]

Supporting Information

Influence of native thin filament type on the regulation of atrial and ventricular myosin motor activity

Emrulla Spahiu[1], Petra Uta[1], Theresia Kraft[1], Arnab Nayak[1*], Mamta Amrute-Nayak[1*]

[1] Institute of Molecular and Cell Physiology, Hannover Medical School, 30625 Hannover, Germany

Corresponding Authors*: Mamta Amrute-Nayak, Arnab Nayak
E-Mail: amrute.mamta@mh-hannover.de
nayak.arnab@mh-Hannover.de

This document contains:
- Figures S1 - S5
- Table S1
- Movie S1

Supplementary Figures

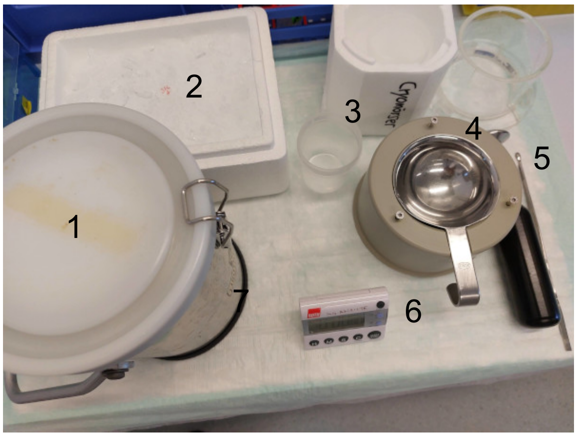

Materials

1. Liquid nitrogen container
2. Ice box and chilled Buffer 1 (skinning)
3. Cups for liquid nitrogen handling
4. Mortar and pestle
5. Steel spatula
6. Timer

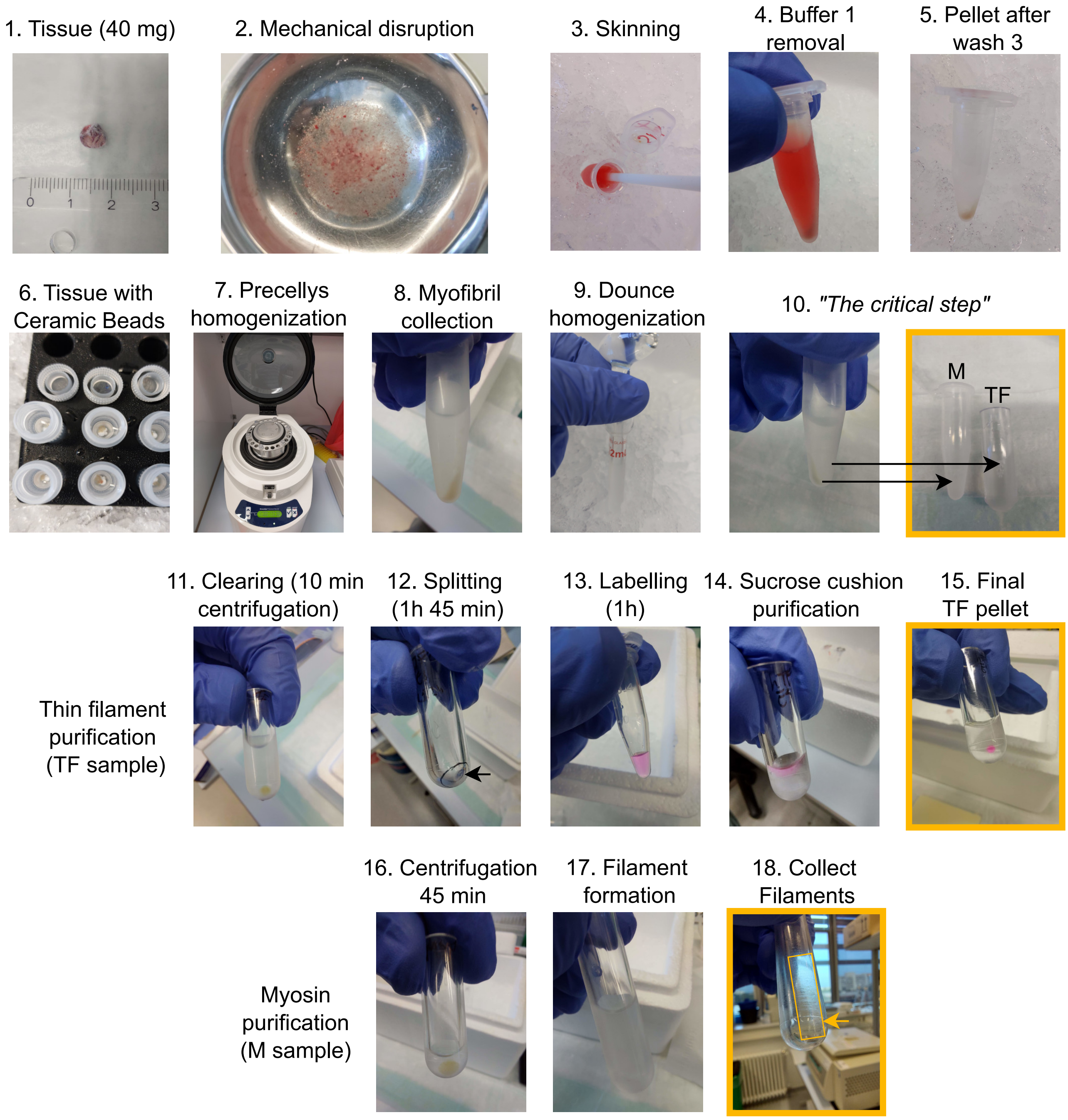

Figure S1. Images from the key steps in the purification protocol

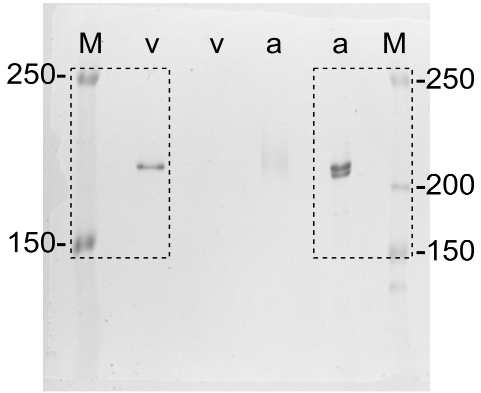

Figure S2. Source gel image of SDS-PAGE used to resolve the myosin isoforms from ventricular (v) and atrial (a) tissues, represented in main Figure 2B. The dashed lines show the crop sites. The marker on the left is Biorad Precision Plus, and the marker on the right is a mix of Biorad Precision Plus (cat.no.1610374) and Thermo Page Ruler (cat.no. 26614).

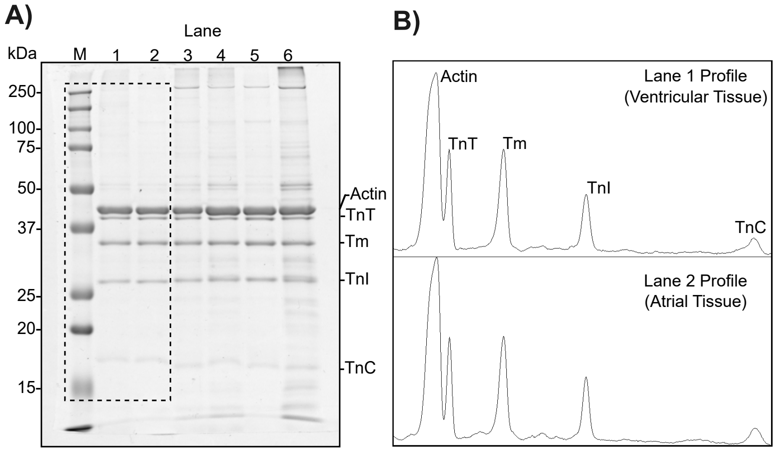

C)

Protein	Uniprot ID	MW (kDa)	Lane (Tissue Source) 1 (v)	2 (a)	3 (v)	4 (a)	5 (v)	6 (a)
**Actin**	G1STB6	42.019	21654	20671	18197	26339	23810	26638
**TnT**	P09741	35.876	4909	4655	4798	4838	6262	5932
**Tm**	P58772	32.681	7790	8301	7595	8948	11046	10217
**TnI**	P02646	24.123	4012	4050	4595	6395	6173	7274
**TnC**	P02591	18.403	1838	1700	1916	2400	2733	2481

Figure S3. Densitometric analysis of thin filament preparations. **A)** 12.5 % SDS-PAGE of six different thin filament preparations from rabbit ventricular (v) and atrial (a) tissue. The protein ladder lane (M) followed by the first two lanes were represented in the main manuscript Figure 2C. **B)** The lane profiles of the first two sample lanes in A) were plotted using the Fiji gel analysis tool. **C)** Pixel intensity values of major thin filament bands for all lanes in A).

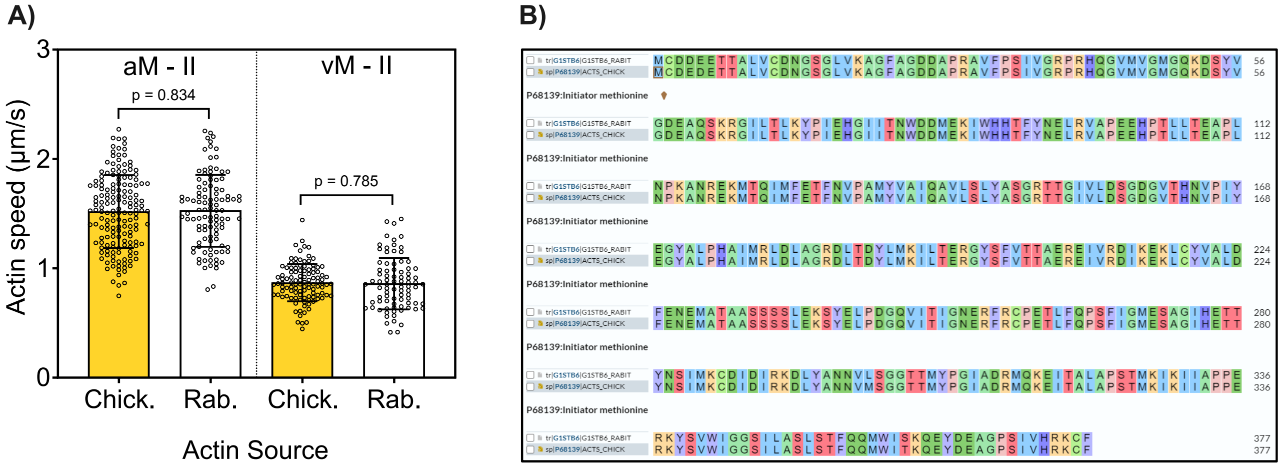

Figure S4. G-actin origin has no impact on motility. **A)** Motility using actin filaments polymerized from G-actin of chicken fast skeletal muscle (Chick.) or rabbit fast skeletal muscle (Rab.) show no significant differences (Mean ± SD, two-tailed unpaired t-test). **B)** Amino acid sequence of alpha skeletal muscle 1 actin from chicken shows 99% similarity to alpha cardiac muscle 1 from rabbit (Uniprot Align Tool).

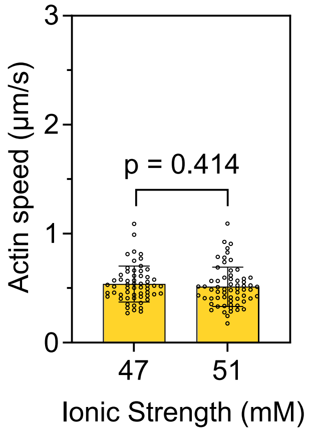

Figure S5. Bar graph showing the effect of elevated ionic strength (4 mM) on native vTF motility driven by native vM-II covered surface at pCa 7.57. An unpaired t-test was used to assess the difference between the two groups having 59-63 filaments.

Supplementary Tables

Table S1. Starting rabbit tissue weight and the final yield of thin filament and myosin samples from the same tissue.

Prep.	Tissue	Tissue (mg)	Thin filament yield		Myosin yield from the same tissue	
			Yield (mg/ml)	μg yield / mg tissue	Yield (mg/ml)	μg yield / mg tissue
1	Ventricle	48	0.66	0.82	3.12	4.86
2	Ventricle	240	2.43	0.61	5.14	1.61
3	Ventricle	228	2.25	0.59	3.75	3.29
4	Ventricle	10	0.55	0.55	0.34	1.30
5	Atrium	127	0.87	0.41	3.05	0.96
6	Atrium	105	0.84	0.48	0.19	0.14
7	Atrium	163	0.38	0.14	0.23	0.10

Supplementary Movie

Movie S1. Representative movie showing native vTF driven on native vM-II motor covered chamber surface. Assay buffer contained 2mM ATP and a pCa 6.48 (332 nM free Calcium) or pCa 5.89 (1.3 μM free Calcium) at a temperature of ~22°C. The original recording was captured at 5 frames per second (fps) and is presented here at playback speed of 50 fps.
